# High-Performance Photoinitiating Systems for LED-Induced Photopolymerization

**DOI:** 10.3390/polym15020342

**Published:** 2023-01-09

**Authors:** Shaohui Liu, Timur Borjigin, Michael Schmitt, Fabrice Morlet-Savary, Pu Xiao, Jacques Lalevée

**Affiliations:** 1 Centre National de la Recherche Scientifique (CNRS), L’Institut de Science des Matériaux de Mulhouse (IS2M UMR 7361), Université de Haute-Alsace, F-68100 Mulhouse, France; 2 Université de Strasbourg, F-67081 Strasbourg, France; 3 Research School of Chemistry, Australian National University, Canberra, ACT 2601, Australia

**Keywords:** photoinitiators, photopolymerization, light emitting diodes

## Abstract

Currently, increasing attention has been focused on light-emitting diodes (LEDs)-induced photopolymerization. The common LEDs (e.g., LED at 365 nm and LED at 405 nm) possess narrow emission bands. Due to their light absorption properties, most commercial photoinitiators are sensitive to UV light and cannot be optimally activated under visible LED irradiation. Although many photoinitiators have been designed for LED-induced free radical polymerization and cationic polymerization, there is still the issue of the mating between photoinitiators and LEDs. Therefore, the development of novel photoinitiators, which could be applied under LED irradiation, is significant. Many photoinitiating systems have been reported in the past decade. In this review, some recently developed photoinitiators used in LED-induced photopolymerization, mainly in the past 5 years, are summarized and categorized as Type Ⅰ photoinitiators, Type Ⅱ photoinitiators, and dye-based photoinitiating systems. In addition, their light absorption properties and photoinitiation efficiencies are discussed.

## 1. Introduction

Monomers can be transformed into polymers under irradiation (e.g., UV and visible light) in photopolymerization. Photopolymerization demonstrates numerous advantages, such as no VOCs, excellent controllability, and high efficiency [1,2,3,4,5]. Currently, photopolymerization is applied in many fields, including coatings [6,7,8], adhesives [9,10,11], 3D printing [12,13,14,15], biomaterials [16,17,18], the microelectronics industry [19,20,21], etc.

Photoinduced polymerization is a chain reaction. Photoinitiators (PIs) can generate active species (free radicals, cations, etc.) under light irradiation to initiate the polymerization of monomers, so a PI has a crucial influence on polymerization rates and final function conversions (FC) for monomers. A photoinitiating system (PIS) can be composed of a PI or PI/additive. Free radical photopolymerization (FRP) can be initiated by generated free radicals. It should be mentioned that FRP is normally inhibited by oxygen, which can quench the primary initiating and propagating radicals. Cationic photopolymerization (CP) is normally sensitive to the presence of moisture [22,23]. Some essential characteristics, including desirable light absorption properties, high photochemical activity, good solubility, and low toxicity, are the important evaluation standard for PIs [24,25,26,27].

Traditional UV light sources (i.e., a mercury lamp) demonstrate many drawbacks, such as short service life, slow switching times, sometimes long heat-up times, and high energy consumption, compared to modern light-emitting diode (LED) light sources. In addition, significant heat generation by broadband illumination of a mercury lamp can affect the surface properties of products, and the ozone release in operation is harmful to the human body [28,29,30,31]. The inherent disadvantages of UV light sources hinder the development of photopolymerization, albeit UV curing is already well-established in the industry. Recently, LEDs have been applied more and more in photopolymerization because of their low cost and high safety [32,33,34]. Nowadays, the emission bands of LEDs are narrow (FWHM in the range of 10 nm) and focused on 365 nm, 395 nm, 405 nm, and 455 nm normally [35]. A large portion of commercial PIs are UV-sensitive and do not absorb light > 360 nm. The effect of the narrow emission bands and the redshift in wavelengths, for example, is clearly presented in the cited literature for different commercial initiators [35]. Therefore, the development of PIs, which can be activated under LED irradiation, is significant [36,37,38,39,40,41,42,43,44,45,46,47].

To match with LEDs, many PISs was designed and developed in the past decade, such as Type Ⅰ and Type Ⅱ PIs, dye-based PISs, metal-complex-based PISs, polyoxometalate-based PISs, and nanoparticle-based PISs, and so on [43,48,49,50,51,52,53,54]. Herein, some highly interesting works for dye-based PISs and the modification of commercial Type Ⅰ PIs and Type Ⅱ PIs are presented. Their light absorption properties are also characterized by their maximal absorption wavelength (λ_max_) and molar absorption coefficient (ε) (see the tables below).

## 2. Dye-Based PISs

Dye molecules have been widely used as PIs in photoinitiation due to their strong absorption in the visible range. Except for dye molecules, the additives, as well as co-initiators, also play important roles in PISs. In the excited states, dye molecules are able to interact with additives through electron transfer reactions to generate active radicals and cations [23,55]. Then, the initial active species can combine with monomers through addition reactions or ring-opening reactions to produce a polymer. Iodonium salts and sulfoniun salts, such as bis(4-tert-butylphenyl)iodonium hexafluorophosphate (Iod) or (sulfanediyldibenzene-4,1-diyl) *bis*(diphenylsulfonium) *bis*(hexafluoroantimonate) in propylene carbonate (Sulf) are frequently used as electron acceptors. Amine compounds, including ethyl 4-(dimethylamino)-benzoate (EDB), N-vinylcarbazole (NVK) and N-phenyl glycine (NPG), are excellent electron donors.

**Scheme 1:**
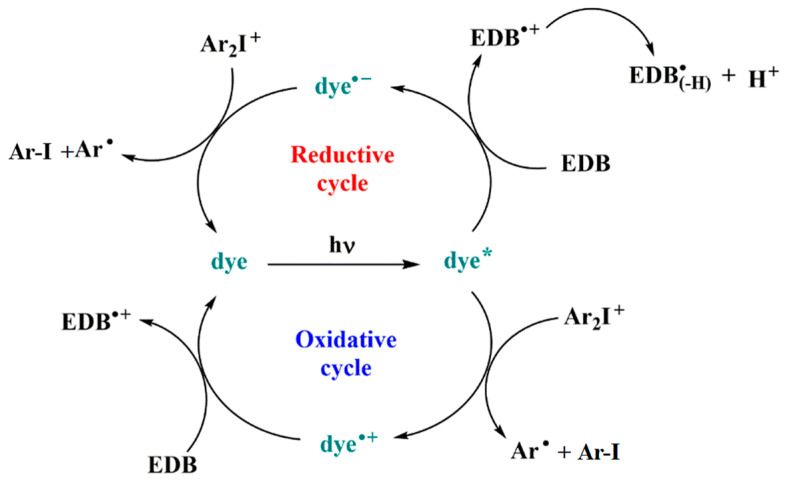
Photochemical mechanism of the dye/Iod/EDB system.

The photochemical mechanism of a typical dye/Iod/EDB system is depicted in Scheme 1. In the reductive cycle, the dye molecule can accept an electron from the electron donor EDB to generate free radical, and the dye**˙**^−^ radical anion is oxidized by Iod to regenerate the dye molecule. In the oxidative cycle, the dye molecule, as an electron donor, transfers an electron to God, producing Ar**˙** and dye**˙+** to induce the FRP and CP, respectively. Then, the dye**˙+** is reduced by EDB to regenerate the dye molecule. The photoredox catalytic cycle slows down the consumption of dye molecules to some degree and accelerates the photopolymerization. Due to their versatile features and good efficiency, numerous dyes have been designed as promising PIs for LED photoinitiation. Here, some representative chemical structures, including carbazole, triphenylamine, naphthalimide, chalcones, and coumarin derivatives, are presented in the following context.

### 2.1. Carbazole-Based Photoinitiators

Carbazole is a representative scaffold applied in the context of an LED photoinitiator. The benzene rings in the carbazole structure can be functionalized with various groups to obtain the extension of conjugation. The nitrogen atom can be modified with alkyl chains to improve solubility. In addition, the remarkable electron-donating ability and low oxidation potential contribute to the interaction with additives in PISs [56,57,58]. The chemical structures of carbazole-based PIs mentioned in this review are shown in Table 1.

A serious of carbazole derivatives were investigated by Mousawi et al. [59,60]. Four carbazole derivatives, C1–C4, were designed as PIs for both FRP and CP. These compounds exhibit good absorptions in the 350–450 nm range. Under irradiation with a LED at 405 nm, high function conversions of 3,4-Epoxycyclohexylmethyl-3′,4′-epoxycyclohexane carboxylate (EPOX) (C1/Iod: FC = 76%, C2/Iod: FC = 50%, C3/Iod: FC = 58%, C4/Iod: FC = 70%) were found for carbazole derivative/Iod combinations. For C/Iod/EDB PISs, a photoredox catalyst behavior could be found in FRP, and the acrylate function conversions for trimethylolpropane triacrylate (TMPTA) were favorable. 3D objects were obtained by a LED projector using these interesting photoinitiating systems. In addition, other carbazole derivatives A1–A4 (Table 1) with thermally activated delayed fluorescence (TADF) properties were synthesized and proposed. Favorable light absorption properties were found for A1–A4, allowing for the application of LED at 405 nm in photopolymerization. High epoxy function conversions (47–55%) were obtained in CP for A/Iod combinations under LED at 405 nm irradiation. Good performances were also observed in the FRP of TMPTA. Finally, these new photoinitiating systems were successfully applied in photocurable 3D printing experiments. Indeed, the TADF property was helpful to the reaction from the excited singlet state between carbazole derivatives and additives.

Four carbazole-based two-photon initiators, A3–1, A3–2, A3–3 and A3–4 (Table 1), containing conjugation bridges were designed and synthesized by Li et al. [61]. Their absorption peaks were located at 350 nm, 360 nm, 415 nm and 394 nm, respectively. No polymerization of TMPTA was found for resin upon exposure to UV, which demonstrated the good stability of carbazole derivatives under one-photon irradiation. The two-photon polymerization using carbazole derivatives was evaluated by direct laser write setup with an 800 nm pulsed laser. In the photopolymerization experiments, A3–2 and A3–4 demonstrated lower threshold energy compared to benchmark PI 1-benzyl-1-(dimethylamino)propyl 4-morpholinophenyl ketone.

**Table 1:** The chemical structures and light absorption properties of carbazole-based photoinitiators.

Chemical Structures	Absorption Properties (λ/nm, ε_max_/M^−1^ cm^−1^)	Refs.
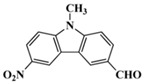 C1	λ_max_ ~ 364 ε_max_ ~ 11,750 ε_405nm_ ~ 2600	[59]
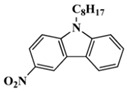 C2	λ_max_ ~ 374 ε_max_ ~ 11,180 ε_405nm_ ~ 5200	[59]
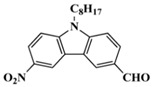 C3	λ_max_ ~ 364 ε_max_ ~ 14,000 ε_405nm_ ~ 2450	[59]
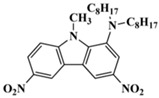 C4	λ_max_ ~ 388 ε_max_ ~ 6000 ε_405nm_ ~ 5200	[59]
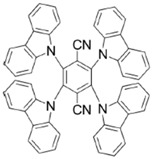 A1	λ_max_ ~ 330 ε_max_ ~ 8800 ε_405nm_ ~ 1350	[60]
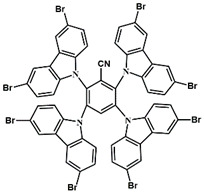 A2	λ_max_ ~ 340 ε_max_ ~ 40,000 ε_405nm_ ~ 7800	[60]
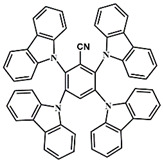 A3	λ_max_ ~ 333 ε_max_ ~ 33,000 ε_405nm_ ~ 5700	[60]
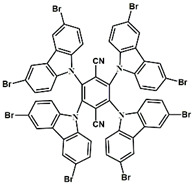 A4	λ_max_ ~ 349 ε_max_ ~ 18,000 ε_405nm_ ~ 3300	[60]
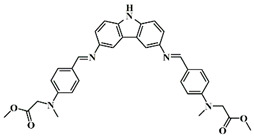 A3–1	λ_max_ ~ 350 ε_max_ ~ 49,000	[61]
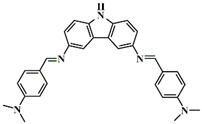 A3–2	λ_max_ ~ 360 ε_max_ ~ 53,000	[61]
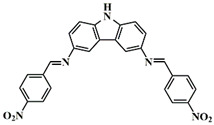 A3–3	λ_max_ ~ 415 ε_max_ ~ 29,000	[61]
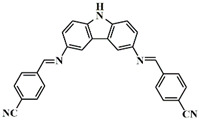 A3–4	λ_max_ ~ 394 ε_max_ ~ 29,000	[61]

### 2.2. Triphenylamine-Based Photoinitiators

Triphenylamine is widely employed for the design of new PIs due to its excellent electron-donating ability. Chemical modification is usually carried out with various groups to obtain extended conjugation. The chemical structures of triphenylamine-based PIs mentioned in this review are given in Table 2. Han et al. [62] synthesized a blue-green-light-sensitive PI CDM2 based on the triphenylamine-curcumin structure. The combination of triphenylamine and curcumin units made it show strong absorption in the blue and green light regions. The introduction of triphenylamine on both sides of the CMD2 reduced the energy gap, which contributed to the red-shifted absorption band. It was very interesting that the maximum absorption wavelength (λ_max_) of CDM2 in tetrahydrofuran was 467 nm, which was suitable for long-wavelength irradiation sources. As a result, the CDM2/iodonium salt system demonstrated good initiation efficiency, and the function conversions of diglycidyl ether of bisphenol A could reach up to 56% and 46% under blue LED or green LED, respectively. 

Two triphenylamine derivatives, Dye3 and Dye4, were designed as PIs by Abdallah et al. [63]. The molar extinction coefficients at 405 nm (ε_405nm_) were 4110 M^−1^ cm^−1^ and 4740 M^−1^ cm^−1^ for Dye3 and Dye4. They were efficient in initiating the CP of EPOX using LED at 405 nm, especially the epoxy function conversion was 66% for Dye3/Iod system. Good performances were shown in FRP of TMPTA for Dye/Iod/NPG PISs, and function conversions were 59% and 57% for Dye3 and Dye4 systems, respectively. The photolysis and fluorescence quenching processes indicated that the reactions for dyes and Iod were effective. Indeed, the electron-donating ability of the triphenylamine core allowed for effective electron transfer reactions.

Three triphenylamine-based hexaarylbiimidazole derivatives (HABI1, HABI2, HABI3) were synthesized by Li [64]. They exhibit favorable absorption bands from 360 nm to 420 nm. The maximum absorption wavelengths (λ_max_) of HABI1–3 were 383, 385, and 384 nm, respectively. The performance of HABIs was evaluated by differential scanning photocalorimetry (photo-DSC) under a UV lamp (250–450 nm) and LED lamp (380–750 nm), respectively. The favorable values of free energy changes proved to have good electron transfer ability for the HABIs/NPG systems. The final function conversions of TMPTA for the HABI1/NPG system were 80% and 58% upon exposure to UV and LED light, respectively. High conversions demonstrated that the HABI1/NPG system could be applied under both UV and LED irradiation sources.

Jin et al. [65] proposed a conjugated sulfonium-based triphenylamine derivative (PI-PAG) as PI in CP. Triarylsulfonium salts are widely used as cationic PI due to the generation of strong protic acids and thermal stability. However, the absorption peaks of most commercial triarylsulfonium salts are below 300 nm, so they cannot be applied under longer-wavelength LEDs, such as LED at 365 and 405 nm. To extend the light absorption band, the sulfonium salt moiety was associated with a long-wavelength triphenylamine chromophore in PI-PAG to form a π-conjugated structure. Interestingly, PI-PAG had good light absorption properties (λ_max_ = 381 nm, ε_365nm_ = 19,200 M^−1^ cm^−1^, ε_405nm_ = 14,900 M^−1^ cm^−1^). The photoinitiation ability of PI-PAG (1 wt%) in CP was evaluated upon exposure to 365, 385, and 405 nm LEDs. The final function conversion for EPOX at 5 min was 52.3% for PI-PAG alone using a LED at 365 nm. When Iod1 was added as a co-initiator, the final function conversion of EPOX reached 82% for PI-PAG/Iod1 (1%/3%, *w*/*w*). In addition, the CP of other monomers, such as cyclohexene oxide and Triethyleneglycol divinyl ether (DVE-3), was also investigated. For PI-PAG (0.5 wt%) alone, the polymerization of DVE-3 was observed. Furthermore, PI-PAG/Iod1 (0.5%/2%, *w*/*w*) had a good performance. Final function conversions of DVE-3 were 90.65% and 90.3% using LED at 365 and 405 nm, respectively.

A photochemical mechanism of PI-PAG was investigated by photolysis and electron spin resonance-spin trapping (ESR-ST) experiments. The S−C bond could break under irradiation; then, the generated sulfur cation radicals further reacted with hydrogen donors to produce the active initiation species H^+^ for CP. The PI-PAG/Iod1 system could also generate cations through an electron transfer reaction. Due to its good performance, PI-PAG successfully initiated the polymerization under chemiluminescence irradiation; the associated chemical mechanisms are presented in detail in [66]. The light emission at ~430 nm was generated through chemical reaction. The CP of cyclohexene oxide was observed in the presence of PI-PAG under the emitted light. These results indicated that the design strategy of π-conjugated sulfonium salts was effective.

**Table 2:** The chemical structures and light absorption properties of triphenylamine-based photoinitiators.

Chemical Structures	Absorption Properties (λ/nm, ε_max_/M^−1^ cm^−1^)	Refs.
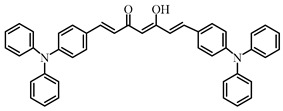 CDM2	λ_max_ ~ 467 ε_max_ ~ 77,190 ε_460nm_ ~ 75,490 ε_520nm_ ~ 12,000	[62]
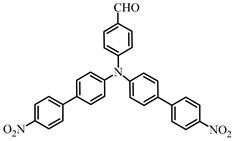 Dye3	λ_max_ ~ 352 ε_max_ ~ 26,610 ε_405nm_ ~ 4110	[63]
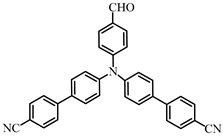 Dye4	λ_max_ ~ 357 ε_max_ ~ 17,700 ε_405nm_ ~ 4740	[63]
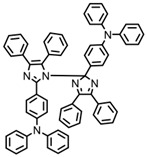 HABI1	λ_max_ ~ 383 ε_max_ ~ 6600	[64]
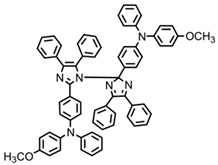 HABI2	λ_max_ ~ 385 ε_max_ ~ 12,100	[64]
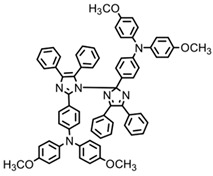 HABI3	λ_max_ ~ 384 ε_max_ ~ 14,800	[64]
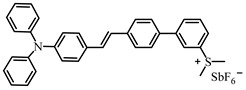 PI-PAG	λ_max_ ~ 381 ε_max_ ~ 23,200 ε_365nm_ ~ 19,200 ε_405nm_ ~ 14,900	[65]

### 2.3. Naphthalimide-Based Photoinitiators

Naphthalimide derivatives are widely used as PIs due to their good stability and ease of synthesis. Additionally, the absorption properties from 400 nm to 600 nm of naphthalimide derivatives can be adjusted by different chemical groups [67,68,69,70]. The chemical structures of naphthalimide-based PIs mentioned in this review are presented in Table 3. A series of naphthalimide derivatives NDP1−NDP7 were proposed as PIs upon LED at 405 nm exposure [71]. The λ_max_ of NDP3 and NDP5 were located below 350 nm. Others demonstrated good light absorption properties because of nitro withdrawing substituent in the naphthalene moiety. Their maximum absorption wavelengths were all located in the range of 417 to 440 nm. The CP of epoxides was evaluated. NDP1/Iod1 and NDP3/Iod1 systems demonstrated low efficiency (FC < 30%) upon exposure to LED at 405 nm. Interestingly, high epoxy function conversions of EPOX 59%, 62% and 63% were obtained in the presence of NDP2/Iod1, NDP4/Iod1, and NDP6/Iod1 systems, respectively, at 800 s. NDP2, NDP4 and NDP6 also exhibit good cationic initiation ability under LED at 455 nm irradiation. The polymerization of TMPTA was evaluated using NDPs/Iod1 and NDPs/Iod1/NVK systems. An excellent performance was observed for NDP2/Iod1 (FC = 52%), NDP2/Iod1/NVK (FC = 63%), NDP4/Iod1 (FC = 52%) and NDP4/Iod1/NVK (FC = 58%). The amino or alkylamino groups contributed to the good light absorption for NDP2, NDP4, and NDP6 structures, which allowed for favorable efficiency in polymerization experiments.

Four naphthalimide derivatives, NDA1–NDA4 with amino or alkylthio substituents, were designed by Xiao et al. [72]. The maximum absorption wavelengths of NDA1, NDA2, NDA3, and NDA4 in acetonitrile are 416 nm, 431 nm, 387 nm, and 439 nm, respectively. Compared to others, the NDA3 exhibited a blue-shifted absorption, which could be attributed to the alkylthio substituent in a naphthalimide skeleton. Steady-state photolysis experiments of NDAs/Iod1 systems exhibit high photochemical reactivity, and favorable electron transfer reaction processes was found. Moreover, the generated phenyl radicals following the NDA2/Iod1 electron transfer reaction were detected in ESR-ST experiments. These naphthalimide derivatives demonstrated good performance in CP, and the epoxy function conversions were all higher than 58% for NDA1/Iod1, NDA2/Iod1, and NDA3/Iod1 systems upon exposure to LED at 405 and 455 nm.

Yu et al. [73] prepared six naphthalimide aryl sulfide derivatives, NAS1–NAS6, and their maximum absorption wavelengths were 389 nm, 385 nm, 340 nm, 387 nm, 391 nm, and 395 nm, respectively. Due to the electron-withdrawing substituents (acetyl, nitro, and fluoro group), blue-shifted absorption was found for NAS2, NAS3, and NAS4, compared to NAS1. The values of free energy changes ΔG for NASs/Iod1 systems were negative, which ensured favorable electron transfer. The CP of the epoxy monomer was carried out for NASs/Iod1 systems using LED at 405 nm. The NAS6/Iod1 system exhibited the best initiation performance, and the epoxy function conversion was 56%. In addition, the C(aryl)-S bond could dissociate to produce active radicals. For different structures, the cleavage of C-S bonds in different positions resulted in the difference in photoinitiation ability. The function conversion of HDDA for NAS6 (0.5 wt%) reached up to 83% upon LED exposure at 405 nm. The electron-rich radicals with isopropyl and methyl groups generated by NAS6 could induce the polymerization of acrylate easily [74]. These naphthalimide aryl sulfide derivatives had high stability under sunlight and demonstrated potential application in the photocuring field.

A PI named Naphth-Iod was designed by Zivic et al. [75]. The λ_max_ of Naphth-Iod was 340 nm, and the absorption band reached up to 390 nm. The CP of EPOX was carried out under LED at 365 nm irradiation. Final epoxy conversions were 40% and 43% in the presence of 1 wt% and 2 wt% Naphth-Iod, respectively, at 800 s. The CP of triethyleneglycol divinyl ether was also carried out on a BaF2 pellet in laminate, and a high vinyl ether function conversion 90% was obtained within 100 s upon exposure to LED at 365 nm at room temperature. In addition, Naphth-Iod could also be used in FRP. The acrylate function conversion of 2,2-bis-[4-(methacryloxy-2-hydroxy-propoxy)-phenyl]-propane (Bis-GMA) and triethylene glycol dimethacrylate (TEGDMA) blend (70/30) was 93% using 2 wt% Naphth-Iod. High-function conversions demonstrated that Naphth-Iod could be used as a versatile PI in CP and FRP. For the photolysis experiment, the absorbance of PI decreased upon exposure to LED at 365 nm. Moreover, the phenyl radical was detected in spin-trapping ESR experiments. Based on these results, a mechanism was proposed. The C-I bond broke to produce a phenyl radical for FRP, and the cation was generated for CP through an in-cage process. In addition, the singlet and triplet excited states energy were higher than the bond dissociation energy (C-I), which was in agreement with the favorable cleavage process.

**Table 3:** The chemical structures and light absorption properties of naphthalimide-based photoinitiators.

Chemical Structures	Absorption Properties (λ/nm, ε_max_/M^−1^ cm^−1^)	Refs.
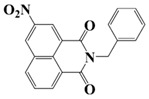 NDP1	λ_max_ ~ 421 ε_max_ ~ 620 ε_405nm_ ~560	[71]
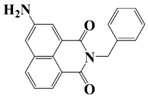 NDP2	λ_max_ ~ 417 ε_max_ ~ 5600 ε_405nm_ ~ 5100	[71]
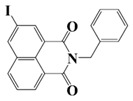 NDP3	λ_max_ ~ 334 ε_max_ ~ 13,100	[71]
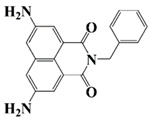 NDP4	λ_max_ ~ 426 ε_max_ ~ 9800 ε_405nm_ ~ 8200	[71]
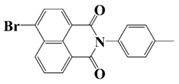 NDP5	λ_max_ ~ 340 ε_max_ ~ 17,800	[71]
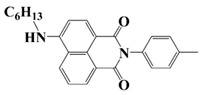 NDP6	λ_max_ ~ 431 ε_max_ ~ 17,400 ε_405nm_ ~ 12,100	[71]
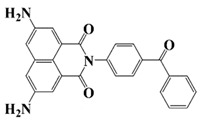 NDP7	λ_max_ ~ 440 ε_max_ ~ 11,300 ε_405nm_ ~ 8800	[71]
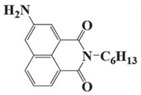 NDA1	λ_max_ ~ 416 ε_max_ ~ 4600 ε_405nm_ ~ 4300 ε_455nm_ ~ 1200	[72]
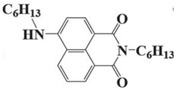 NDA2	λ_max_ ~ 431 ε_max_ ~ 14,600 ε_405nm_ ~ 10,300	[72]
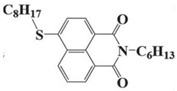 NDA3	λ_max_ ~ 387 ε_max_ ~ 18,000 ε_405nm_ ~ 13,000 ε_455nm_ ~ 1000	[72]
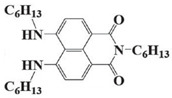 NDA4	λ_max_ ~ 439 ε_max_ ~ 16,300 ε_405nm_ ~ 9300	[72]
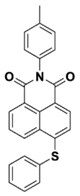 NAS1	λ_max_ ~ 389 ε_405nm_ ~ 10,100	[73]
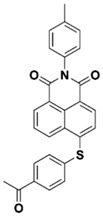 NAS2	λ_max_ ~ 385 ε_405nm_ ~ 9300	[73]
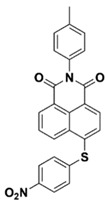 NAS3	λ_max_ ~ 340 ε_405nm_ ~ 6100	[73]
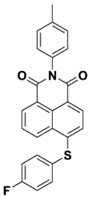 NAS4	λ_max_ ~ 387 ε_405nm_ ~ 12,800	[73]
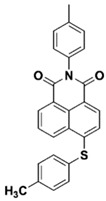 NAS5	λ_max_ ~ 391 ε_405nm_ ~ 12,600	[73]
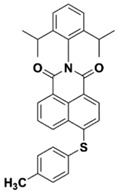 NAS6	λ_max_ ~ 395 ε_405nm_ ~ 11,700	[73]
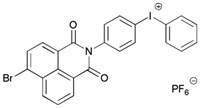 Naphth-Iod	λ_max_ ~ 340	[75]

### 2.4. Coumarine-Based Photoinitiators

Coumarin derivatives are widely applied in many fields, such as fluorescent bio labels and organic light-emitting diodes. Through chemical modification, a wide variety of electronic, photochemical and photophysical properties can be obtained for coumarin derivatives [76]. Therefore, coumarin is a good scaffold to use in the design of PIs. The chemical structures of coumarin-based PIs mentioned in this review are shown in Table 4. Two coumarin derivatives (CoumA and CoumB) were designed by Abdallah et al. [77]. Good light absorption (CoumA: λ_max_ = 421 nm; CoumB: λ_max_ = 405 nm) ensured the availability of LED at 405 nm. The polymerization of EPOX (25 μm) in the air was studied using LED at 405 nm. A fast polymerization rate and high epoxy function conversion (FC = 80%) were found for the CoumA/Iod system. However, no polymerization of EPOX was observed for the CoumB/Iod system in the same condition. The acrylate function conversions of TMPTA were 81% and 93% for the CoumA/Iod/NPG and CoumB/Iod/NPG systems. Efficient interactions between coumarins and Iod were observed. Indeed, coumarin derivatives as photoredox catalysts were efficient when amine was used as an electron donor and iodonium salt as an electron acceptor. In addition, water-soluble CoumB was also used for hydrogel synthesis. Finally, free radical polymerization in 3D printing was successfully proved due to the high performance of CoumA and CoumB.

Some derivatives bearing an iodonium salt moiety were designed as efficient one-component PIs by Topa et al. [78]. Their maximum absorption wavelengths were 350 nm. The polymerization of EPOX was evaluated using coumarin derivatives alone under air. 7M-P demonstrated the best initiation ability among them, and the epoxy function conversions were 58% and 53% using LED at 365 and 405 nm, respectively. Due to the good performance of coumarin-based PIs in CP and FRP, 3D-printed objects were fabricated successfully using TMPTA/EPOX blends. In steady-state photolysis experiments, the absorbance of coumarin derivatives at maximum absorption wavelength decreased obviously under LED at 365 and 405 nm irradiation. According to laser flash photolysis (LFP) experiments, the iodonium salts dissociated by homolytic or heterolytic cleavage of carbon–iodine bond. The BDE of coumarin moiety-iodine was higher than that of I-Ph-R bond. It indicated the cleavage in I-Ph-R moiety was more favorable. In addition, the aryl radicals were detected by spin-trapping ESR experiments. Based on the above results, a cleavage mechanism was proposed.

**Table 4:** The chemical structures and light absorption properties of coumarin-based photoinitiators.

Chemical Structures	Absorption Properties (λ/nm, ε_max_/M^−1^ cm^−1^)	Refs.
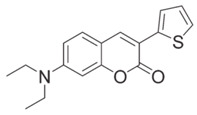 CoumA	λ_max_ ~ 421 ε_max_ ~ 35,200 ε_405nm_ ~ 30,600	[77]
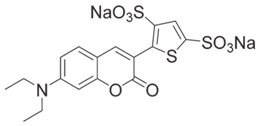 CoumB	λ_max_ ~ 405 ε_max_ ~ 28,100 ε_405nm_ ~ 28,100	[77]
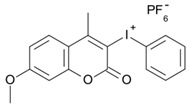 7M-P	λ_max_ ~ 350 ε_max_ ~ 20,440 ε_365nm_ ~ 16,660 ε_405nm_ ~ 280	[78]
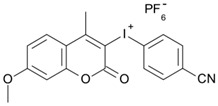 7M-CN-P	λ_max_ ~ 352 ε_max_ ~ 18,930 ε_365nm_ ~ 16,800 ε_405nm_ ~ 360	[78]
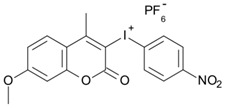 7M-NO_2_-P	λ_max_ ~ 351 ε_max_ ~ 19,100 ε_365nm_ ~ 16,770 ε_405nm_ ~ 460	[78]
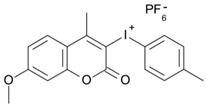 7M-Me-P	λ_max_ ~ 349 ε_max_ ~ 22,700 ε_365nm_ ~ 18,200 ε_405nm_ ~ 240	[78]
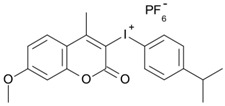 7M-iPr-P	λ_max_ ~ 350 ε_max_ ~ 18,440 ε_365nm_ ~ 14,960 ε_405nm_ ~ 220	[78]

### 2.5. Chalcone-Based Photoinitiators

Chalcone is a natural scaffold that has been found in numerous plants. Chalcones have been applied in many fields, including organogels and organic photovoltaics [79]. Interestingly, chalcones can be synthesized directly by condensation of an aldehyde with acetophenone [80,81]. Markedly, chalcones also undergo a competing [2+2] photodimerization, which can be employed for photocrosslinking. Therefore, as an environment-friendly and available dye, chalcone has been employed to design bioinspired PIs in recent years [82,83,84]. The chemical structures of chalcone-based PIs mentioned in this review are shown in Table 5.

A series of chalcones bearing carbazole or triphenylamine moiety was designed by Chen et al. [85]. Carbazole or triphenylamine were outstanding electron donors. The combination of carbazole or triphenylamine with chalcone formed D-π-A or A-π-D-π-A structures. Except for Chalcones 2, 3, 5, and 6, λ_max_ of all the compounds were located in the visible region (λ > 400 nm). Particularly, high molar extinction coefficients were observed for Chalcone 4, Chalcone 7 and Chalcone 10. PEG-diacrylate (SR 610) was used in FRP under LED at 405 nm irradiation. Chalcones/Iod and Chalcones/Iod/EDB systems exhibit favorable efficiency in thick molds (2 mm) and thin films (0.1 mm). Chalcone 4, 7, and 10 exhibited the best performance in Chalcones/Iod and Chalcones/Iod/EDB systems. High acrylate function conversions of PEG-diacrylate in thick molds were observed for Chalcone 4/Iod/EDB (FC = 95%), Chalcone 7/Iod/EDB (FC = 94%), and Chalcone 10/Iod/EDB (FC = 92%). 3D-printed patterns were successfully obtained for Chalcones/Iod systems through laser write experiments. In addition, the shapes of 3D patterns using the Chalcone 7/Iod system had reversible deformation behavior because of the hydrophilic response.

A series of bis-chalcone compounds were proposed by Chen et al. [86]. These bis-chalcone compounds exhibit favorable absorption. High ε_405nm_ for Bis-chalcone 5 and Bis-chalcone9 were observed. The FRP of PEG-acrylate monomer in laminate (~20 μm) was evaluated using bis-chalcone/Iod/EDB systems upon exposure to LED at 405 nm. The structures Bis-chalcone 5, 7, and 9 exhibited good initiation ability, and the acrylate function conversions were 62%, 95%, and 91%, respectively. Due to the high photoactivity, 3D-printed objects were successfully fabricated in laser write experiments (laser diode at 405 nm) using Bis-chalcone 5 and 9. In addition, other chalcone compounds were synthesized by Chen et al. [87,88]. These studies provide a powerful tool for designing the PISs used in LED photopolymerization.

**Table 5:** The chemical structures and light absorption properties of chalcone-based photoinitiators.

Chemical Structures	Absorption Properties (λ/nm, ε_max_/M^−1^ cm^−1^)	Refs.
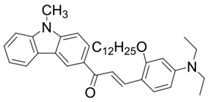 Chalcone 1	λ_max_ ~ 425 ε_max_ ~ 8930 ε_405nm_ ~ 7450	[85]
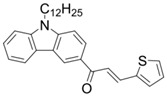 Chalcone 2	λ_max_ ~ 369 ε_max_ ~ 20,520 ε_405nm_ ~ 4830	[85]
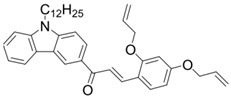 Chalcone 3	λ_max_ ~ 369 ε_max_ ~ 17,740 ε_405nm_ ~ 5960	[85]
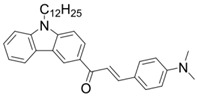 Chalcone 4	λ_max_ ~ 408 ε_max_ ~ 23,900 ε_405nm_ ~ 23,580	[85]
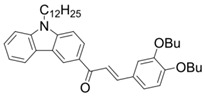 Chalcone 5	λ_max_ ~ 370 ε_max_ ~ 21,100 ε_405nm_ ~ 7020	[85]
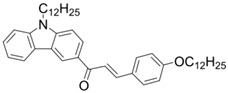 Chalcone 6	λ_max_ ~ 360 ε_max_ ~ 24,900 ε_405nm_ ~ 3530	[85]
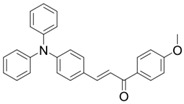 Chalcone 7	λ_max_ ~ 405 ε_max_ ~ 18,740 ε_405nm_ ~ 18,740	[85]
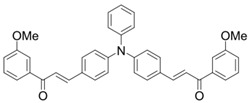 Chalcone 8	λ_max_ ~ 430 ε_max_ ~ 7990 ε_405nm_ ~ 6760	[85]
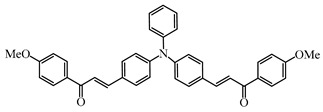 Chalcone 9	λ_max_ ~ 428 ε_max_ ~ 8540 ε_405nm_ ~ 7200	[85]
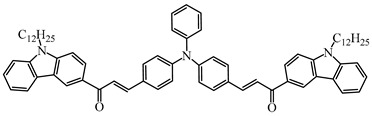 Chalcone 10	λ_max_ ~ 430 ε_max_ ~ 10,500 ε_405nm_ ~ 9020	[85]
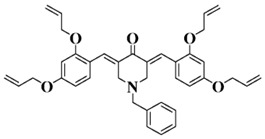 Bis-chalcone 1	λ_max_ ~ 370 ε_max_ ~ 21,900 ε_405nm_ ~ 12,740	[86]
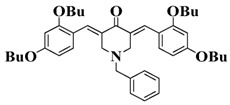 Bis-chalcone 2	λ_max_ ~ 380 ε_max_ ~ 28,200 ε_405nm_ ~ 19,730	[86]
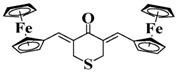 Bis-chalcone 3	λ_max_ ~ 332 ε_max_ ~ 18,200 ε_405nm_ ~ 4420	[86]
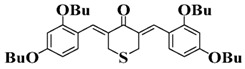 Bis-chalcone 4	λ_max_ ~ 369 ε_max_ ~ 4980 ε_405nm_ ~ 2550	[86]
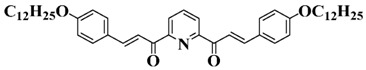 Bis-chalcone 5	λ_max_ ~ 347 ε_max_ ~ 23,100 ε_405nm_ ~ 6800	[86]
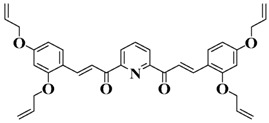 Bis-chalcone 6	λ_max_ ~ 364 ε_max_ ~ 22,700 ε_405nm_ ~ 10,070	[86]
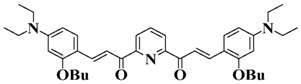 Bis-chalcone 7	λ_max_ ~ 430 ε_max_ ~ 38,900 ε_405nm_ ~ 26,420	[86]
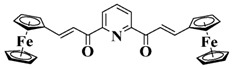 Bis-chalcone8	λ_max_ ~ 330 ε_max_ ~ 19,800 ε_405nm_ ~ 2960	[86]
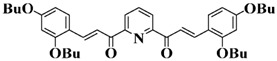 Bis-chalcone 9	λ_max_ ~ 370 ε_max_ ~ 24,600 ε_405nm_ ~ 13,670	[86]
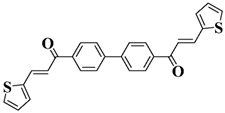 Bis-chalcone 10	λ_max_ ~ 350 ε_max_ ~ 49,300 ε_405nm_ ~ 2220	[86]

## 3. Type Ⅱ Photoinitiators

Many studies have focused on Type Ⅱ PIs which can interact with hydrogen donors through H-abstraction reaction to generate two free radicals. Some ketone-type compounds, including benzophenone (BP), thioxanthone (TX), and camphorquinone (CQ), are widely used as Type II PIs. Tertiary amines and thiols are normally used as hydrogen donors due to their good reductant behavior [89,90]. After electron transfer and proton transfer, two free radicals are generated. Because of steric hindrance and delocalization of unpaired electrons, ketyl radicals from ketone compounds have no reactivity to double bonds [91]. The absorption for most traditional Type Ⅱ PIs are mainly located in the UV region, so many efforts have been devoted to expanding the light absorption range of Type Ⅱ PIs to match the near UV or visible LEDs. Introducing the Type Ⅱ PI structures onto dye scaffolds to obtain π-conjugated structures is an effective method. The π-conjugated structure is expected to expand the absorption and achieve good H-abstraction ability. The obtained structures can be classified as dye-based PISs. However, compared to the general dye molecules, the obtained structures demonstrate a more obvious feature of Type Ⅱ PI in the presence of hydrogen donors. Therefore, these structures are separately listed as Type Ⅱ PIs in this review. Benzophenone and thioxanthone are the most commonly employed, and some published chemical structures are introduced in the following context.

### 3.1. Benzophenone Photoinitiators

As a traditional UV-sensitive PI, BP does not absorb light in longer wavelength regions (>380 nm). BP was modified widely to obtain better photoinitiation ability. Many groups, including amino, thioether, and oxime, were induced into the BP skeleton, and these BP derivatives demonstrated good efficiency under UV light or halogen lamp [92,93,94,95,96,97,98,99,100,101,102]. Herein, the BP derivatives proposed in recent years are mainly summarized. The chemical structures of benzophenone PIs mentioned in this review are presented in Table 6.

A Type Ⅱ PI (Py_BP) based on the BP group linked to a pyrene moiety was designed by Tehfe et al. [103]. The maximum absorption wavelength of Py_BP was 348 nm, and high values of ε_348nm_ = 26,000 M^−1^ cm^−1^, and ε_405nm_ = 1400 M^−1^ cm^−1^ were found. The FRP of TMPTA was carried out upon halogen lamp exposure (λ > 300 nm), and no polymerization was found for BP/amine system. The function conversion of TMPTA reached up to 35% using Py_BP/*N*-methyldiethanolamine (MDEA) (1%/4%, wt%/wt%) system. Indeed, the good light absorption properties and H-abstraction ability allowed an effective Type Ⅱ behavior for Py_BP. A good polymerization profile (FC = 55% at 800 s) was also observed in the CP of EPOX upon halogen lamp exposure for Py_BP/Iod (0.5%/2%, wt%/wt%) system. All results indicated that Py_BP was a reactive PI for both FRP and CP. Another BP derivative (Py_BP5) with the coupling of BP and pyrene moieties was designed by Telitel et al. [104]. Py_BP5 had a hybrid structure where BP and pyrene were fused via a benzene ring. The maximum absorption wavelength of Py_BP5 was 395 nm. Compared to Py_BP, Py_BP5 had a red-shifted absorption wavelength which could be attributed to the strong molecular orbital coupling. Free radical polymerization was carried out under Xe-Hg lamp with a filter (λ > 340 nm) irradiation. The function conversion of 40% of TMPTA was obtained using Py_BP5/MDEA (1%/2%, wt%/wt%) system at t = 200 s. Indeed, combining BP with a light-absorbing moiety is a good method to improve the light absorption properties of BP derivatives, and it was also reported in other works [105].

Zhang et al. [106] connected the BP group with naphthalimide chromophore to design another Type Ⅱ PI BPND. Good light absorption properties (λ_max_ = 431 nm, ε_405nm_ = 15,700 M^−1^ cm^−1^) were obtained. In photolysis experiments, the absorbance of BPND at 431 nm decreased slowly during the irradiation at 405 nm, on account of the H-abstraction for two BPND molecules probably. The generated aminoalkyl radical was detected in spin-trapping ESR experiments. When Iod was added, fast bleaching was observed under irradiation. In FRP experiments, BPND alone could initiate the polymerization of TMPTA as one-component Type Ⅱ PI. Interestingly, BPND/Iod system exhibit excellent and similar photoinitiation ability (55~57%) under LED at 405, 455 and 470 nm irradiation. High function conversions (56~70%) were also obtained in CP for EPOX in the presence of the BPND/Iod system. The high performance of BPND benefits from the good design of the molecular structure. In a word, the ketone-dye-based compounds demonstrate great potential in the design of PIs for LED photoinitiation.

Five visible light benzophenone-based PIs (BP1–BP5) were synthesized by Huang et al. [107]. In these structures, BP was incorporated into different arylamin moieties. The UV absorption spectra of BPs were investigated in a dichloromethane solution. Compared to BP, BP derivatives exhibit red-shifted absorption, and λ_max_ of them were all located in a range of 360 to 375 nm. Due to the good absorption properties and the BP moiety, these PIs were expected to act as efficient visible Type Ⅱ PIs in FRP. The photolysis of BPs/triethylamine (TEA) systems were studied under 365 nm light irradiation. The absorption band at ~360 nm decreased; meanwhile, an increasing peak at ~290 nm was observed. The FRP of TMPTA under a UV lamp was evaluated by photo-DSC in the presence of BPs/TEA systems. The reference BP/TEA system demonstrated the best performance among them. No polymerization was observed for BP3/EDB system. The polymerization of TMPTA was also evaluated under white light LED irradiation (380–750 nm). High function conversions of TMPTA were found for BP-1/TEA and BP/TEA systems, respectively. Indeed, good light absorption properties ensured the efficient photoinitiation ability for BP-1 as a Type Ⅱ PI.

Three compounds named C-DBP, P-DBP and T-DBP were designed as PIs for LED photopolymerization by Jia et al. [108]. These chemical structures incorporated benzophenone as an electron acceptor and carbazole/phenothiazine/triphenylamine as an electron donor. Considering the photo-isomerization of the double bond, the triple bond is a desired π-linker for D-π-A structures to extend the conjugated structures. These PIs had favorable absorption bands in the region of 300 to 450 nm. The FRP of tripropylene glycol diacrylate (TPGDA) were carried out upon exposure to LED at 405 nm using DBP/triethanolamine (TEOA) system. It was interesting that C-DBP/TEOA (0.25%/2%, *w*/*w*) exhiexhibitedd photoinitiation ability and the acrylate function conversion reached up to 95%, while P-DBP and T-DBP systems demonstrated poor performance in polymerization experiments. In fluorescence and nanosecond transient absorption experiments, C-DBP demonstrated both BP-like features, contributing to good H-abstraction ability. Thus, C-DBP had good performance in free radical photopolymerization.

A benzophenone derivative named BPN was designed by Xue et al. [109]. BPN had strong adsorption in the range of 320~500 nm (ε_405nm_ = 42,400 M^−1^ cm^−1^), indicating BPN could be used as visible light PI under LED illumination. It was interesting that the polymerization of the TPGDA blends were observed at LED at 405 nm without any additional co-initiator. The acrylate function conversion was 80% at 80 s in the presence of 0.5 wt% BPN. It was attributed to dimethyl amine moiety in the structure. Interestingly, the faster polymerization rate and higher function conversion (90%) were obtained using BPN/TEOA system. BPN demonstrated the same performance as benchmark ITX in polymerization experiments. Fast photolysis was observed for BPN alone upon exposure to LED at 405 nm. The reason was that an H-abstraction reaction occurred between benzophenone and amine groups in two BPN molecules. In addition, the absorption of BPN declined fast in the presence of TEOA. Indeed, the H-abstraction reaction mainly occurred in BPN/TEOA bimolecular system. In spin-trapping ESR experiments, the generated BPN_(-H)_ radical and TEOA_(-H)_ radical were detected, which verified the H-abstraction reaction in unimolecular and bimolecular systems, respectively. Because of its high performance, BPN has great potential to be used in photoinitiation.

A series of benzophenone-carbazole compounds (BPC-BPC4) was investigated by Liu et al. [110,111]. Interestingly, the benzoyl substituent is connected with carbazole to generate a benzophenone moiety. The benzophenone-carbazole structure demonstrates the feature of the Type Ⅱ photoinitiator (PI). Good absorption in the UV region was found (ε_max_ = 18,600 M^−1^ cm^−1^, λ_max_ = 342 nm) for BPC and the value of ε_365nm_ was favorable. Therefore, their photoinitiation performances of them were studied using a LED at 365 nm. Benzophenone-carbazole PIs could initiate the polymerization of monomers alone. In addition, better performances were found for two-component systems (FC = 60% for BPC/EDB system; FC = 63% for BPC/Iod system). Besides, these PIs demonstrated favorable performance in cationic polymerization of EPOX (FC = 46% for BPC/Iod system).

Some benzophenone-triphenylamine PIs (BT1–BT4) were designed for LED-induced photopolymerization [112]. Interestingly, benzophenone-triphenylamine PIs exhibit a favorable molar extinction coefficient at 405 nm (e.g., ε_405nm_ (BT3) = 6100 M^−1^ cm^−1^, ε_405nm_ (BT4) = 6700 M^−1^ cm^−1^). Some PIs demonstrated better performances than benchmark isopropylthioxanthone (ITX) in free radical polymerization upon exposure to LED at 405 nm. In cationic polymerization, good polymerization profiles of EPOX were found. Finally, 3D-printed objects were obtained successfully using the developed new PIs, and these chemical structures demonstrated rational design for PIs [112,113].

**Table 6:** The chemical structures of benzophenone photoinitiators and their absorption properties.

Chemical Structures	Absorption Properties (λ/nm, ε_max_/M^−1^ cm^−1^)	Refs.
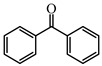 BP	λ_max_ ~ 253 ε_max_ ~ 22,000 ε_363nm_ ~250	[104,107]
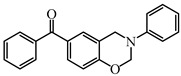 BPB	λ_max_ ~ 290 ε_max_ ~ 14,610	[100]
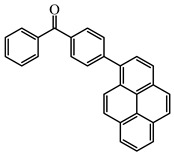 Py_BP	λ_max_ ~ 348 ε_max_ ~ 26,000 ε_405nm_ ~1400	[103]
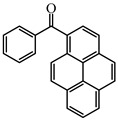 Py_BP5	λ_max_ ~ 395 ε_max_ ~ 4400	[104]
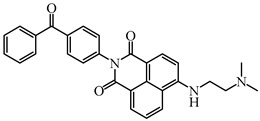 BPND	λ_max_ ~ 431 ε_max_ ~ 15,700 ε_405nm_ ~ 10,900 ε_470nm_ ~ 5700	[106]
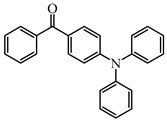 BP1	λ_max_ ~ 369 ε_max_ ~ 22,300	[107]
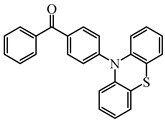 BP2	λ_max_ ~ 341 ε_max_ ~ 13,300	[107]
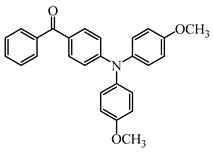 BP3	λ_max_ ~ 368 ε_max_ ~ 20,200	[107]
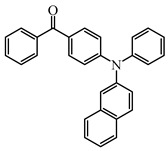 BP4	λ_max_ ~ 369 ε_max_ ~ 42,900	[107]
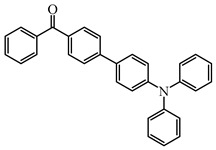 BP5	λ_max_ ~ 374 ε_max_ ~ 27,800	[107]
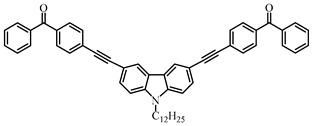 A4	λ_max_ ~ 349 ε_max_ ~ 18,000	[108]
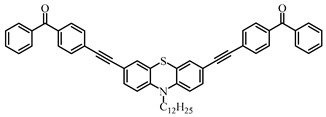 A3–1	λ_max_ ~ 350 ε_max_ ~ 49,000	[108]
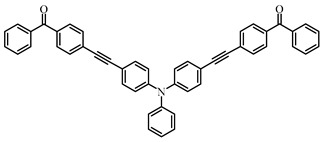 A3–2	λ_max_ ~ 360 ε_max_ ~ 53,000	[108]
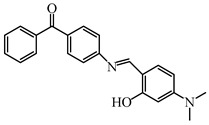 BPN	λ_max_ ~ 400 ε_max_ ~ 43,700 ε_405nm_ ~ 42,400	[109]
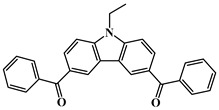 BPC	λ_max_ ~ 342 ε_max_ ~ 18,600 ε_365nm_ ~ 6000	[110]
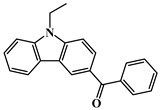 BPC1	λ_max_ ~ 334 ε_max_ ~ 13,910 ε_365nm_ ~ 3270	[111]
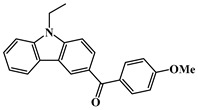 BPC2	λ_max_ ~ 325 ε_max_ ~ 13,900 ε_365nm_ ~ 2210	[111]
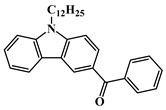 BPC3	λ_max_ ~ 334 ε_max_ ~ 13,350 ε_365nm_ ~ 3460	[111]
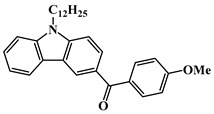 BPC4	λ_max_ ~ 325 ε_max_ ~ 12,400 ε_365nm_ ~ 2170	[111]
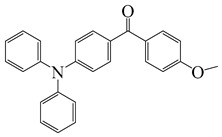 BT1	λ_max_ ~ 359 ε_max_ ~ 21,000 ε_405nm_ ~ 1800	[112]
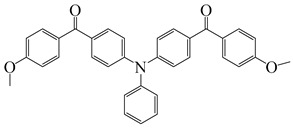 BT2	λ_max_ ~ 373 ε_max_ ~ 27,200 ε_405nm_ ~ 5000	[112]
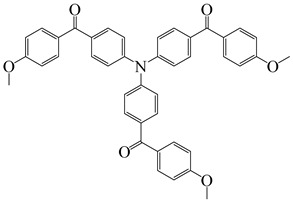 BT3	λ_max_ ~ 370 ε_max_ ~ 41,600 ε_405nm_ ~ 6100	[112]
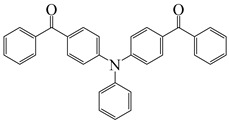 BT4	λ_max_ ~ 377 ε_max_ ~ 21,700 ε_405nm_ ~ 6700	[112]

### 3.2. Thioxanthone Photoinitiators

Thioxanthone (TX) is another efficient Type Ⅱ PI, and the TX derivatives are widely used in FRP and CP. Compared to BP, TX exhibits a longer absorption wavelength in the range of 300–400 nm, so TX derivatives are in a class of potential visible light PIs [114,115,116]. As known, the commercial isopropylthioxanthone (ITX) is widely used as a benchmark Type Ⅱ PI. The chemical structures of thioxanthone PIs mentioned in this review are given in Table 7.

A thioxanthone-carbazole derivative (TX-C) was synthesized by Yilmaz et al. [117,118,119]. TX moiety was in conjunction with carbazole chromophore to obtain light absorption in the visible range. TX-C demonstrated good absorption at a longer wavelength range (400–500 nm), where TX was almost transparent. Indeed, the extended conjugation of TX and carbazole moieties contributed to the longer absorption wavelength. The λ_max_ was 434 nm for TX-C, and a high molar extinction coefficient was found (ε_434nm_ = 2010 M^−1^ cm^−1^). In the steady-state photolysis experiments, the absorbance at 434 nm of TX-C decreased. It indicated that an H-abstraction reaction occurred between TX and the amine group in the carbazole moiety. Therefore, TX-C could be used as a one-component Type Ⅱ PI without an additional hydrogen donor. The initiation ability of PI was evaluated upon exposure to visible light (430–490 nm). There is no polymerization of monomers in the presence of the TX/amine system due to the poor absorption property of TX in the visible range. As expected, TX-C could initiate the polymerization of methyl methacrylate (MMA) alone. The excited state TX-C reacted with the ground state TX-C to generate carbazoyl radical, which was active in the polymerization of MMA. When the amine (hydrogen donor) was added, TX-C/amine system demonstrated a faster polymerization rate than one-component TX-C. Indeed, in the presence of amine, H-abstraction occurred easily between TX-C and amine. In summary, TX-C was an excellent visible Type Ⅱ PI and had many targeted applications.

A thioxanthone-anthracene PI (TX-A) was synthesized by Balta et al. [120,121]. The maximum absorption wavelength of TX-A was 368 nm and the absorption band extended to 450 nm. In photolysis experiments, the absorption band of the anthracene moiety almost disappeared with the consumption of TX-A, while the absorption of thioxanthone moiety at 380 nm was still observed. TX-A was used as a PI for the FRP of MMA. It was strange that higher function conversions were found in the presence of TX-A under air, compared to TX-A/amine system. In addition, no polymerization was found under nitrogen. Based on the photolysis and polymerization experiments, the conclusions could be obtained that TX moiety did not likely participate in the H-abstraction reaction, and oxygen played an important role in polymerization. As known, when the triplet oxygen (ground state molecular oxygen) is quenched, singlet oxygen will produce through energy transfer. In an air-saturated TX-A solution, the generation of singlet oxygen was confirmed by the NIR luminescence spectrum. Therefore, singlet oxygen could react with anthracene moiety to generate endoperoxide. Then endoperoxide decomposed into radicals that were active for monomers. The chemical mechanism of TX-A is helpful in overcoming oxygen inhibition in FRP processes [122,123].

A PI named TX-NPG was designed by Tar et al. [124]. TX-NPG exhibit good light absorption in the region of 300–600 nm in *N*,*N*-dimethylformamide. Favorable molar extinction coefficients at 392 nm (ε_392nm_ = 1670 M^−1^ cm^−1^) and 583 nm (ε_583nm_ = 440 M^−1^ cm^−1^) were observed. The red-shifted band (~583 nm) disappeared when triethylamine was added to the solution, which could be attributed to the intramolecular or intermolecular hydrogen bond in TX-NPG solution. This peculiar absorption was also reported in other TX derivatives, such as thioxanthone carboxylic acid and sodium fluorenecarboxylate-thioxanthone [115,125]. Favorable absorption properties made TX-NPG attractive as a visible PI. TX-NPG was investigated as a Type Ⅱ PI in the photopolymerization experiments under irradiation with 392, 473, 532, and 635 nm. In the presence of TX-NPG alone, the function conversions of acrylate monomers under irradiation at 392, 473, 532, and 635 nm were 63%, 21%, 24% and 14%, respectively. This was attributed to the H-abstraction reaction in a one-component system. The addition of MDEA as a hydrogen donor contributed to the H-abstraction reaction, and higher final function conversions were obtained. The performance of TX-NPG demonstrated the possibility of its use under panchromatic irradiation. In addition, several TX derivatives bearing abstractable hydrogen sites (amine group) were also designed as one-component PIs [126,127,128].

Three TX derivatives (TX-2CBZ, TX-2DPA, TX-2PTZ) with D-A-D structures were designed by Mau et al. [129]. In the D-A-D structures, the TX moiety acted as the acceptor and dimethoxyphenylamine, carbazole, phenothiazine moieties acted as electron donors, respectively. These D-A-D structures were expected to obtain enhanced light absorption. Three investigated TX derivatives exhibited a bathochromic shift (λ_max_ = 396 nm for TX-2CBZ; λ_max_ = 478 nm for TX-2DPA; λ_max_ = 415 nm for TX-2PTZ) compared to ITX (λ_max_ = 386 nm). Meanwhile, three compounds demonstrated higher extinction coefficients at 405 nm (ε_405nm_ = 5900 M^−1^ cm^−1^ for TX-2CBZ; ε_405nm_ = 2000 M^−1^ cm^−1^ for TX-2DPA; ε_405nm_ = 2600 M^−1^ cm^−1^ for TX-2PTZ) than ITX (ε_405nm_ = 1000 M^−1^ cm^−1^). These PIs were evaluated as Type Ⅱ PIs in the presence of EDB. TX-2CBZ and ITX exhibited good performances in the polymerization of TMPTA, and function conversions were 83% and 84%, respectively. Furthermore, poor efficiencies were found in the presence of TX-2DPA and TX-2PTZ. 

To explore the relationships between structure and efficiency, the singlet and triplet states of TX derivatives were detected. The results showed that a thioxanthone triplet state was found in TX-2CBZ and ITX structures, and the lifetime were about 1.0 μs and 5.8 μs, respectively. TX-2CBZ demonstrated strong H-abstraction ability due to the TX moiety in structure, while no significant H-abstraction was observed for TX-2DPA and TX-2PTZ. It could possibly be ascribed to the internal conversion as a deactivation pathway of the excited state, which resulted in the slow reaction with EDB. The TX-2CBZ/Iod system also exhibited good performance in the CP of EPOX (FC = 59%). In the 3D-printing experiments, the pattern was successfully fabricated using a three-component TX-2CBZ/Iod/EDB system with TMPTA/EPOX blend. The PI TX-2CBZ, which had a similar performance to ITX, was applied in 3D printing [130].

**Table 7:** The chemical structures of thioxanthone photoinitiators and their absorption properties.

Chemical Structures	Absorption Properties (λ/nm, ε_max_/M^−1^ cm^−1^)	Refs.
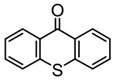 TX	λ_max_ ~ 380 ε_max_ ~ 5300	[131]
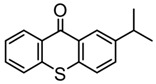 ITX	λ_max_ ~ 386 ε_max_ ~ 6500 ε_395nm_ ~ 3900	[132]
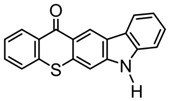 TX-C	λ_max_ ~ 434 ε_max_ ~ 2010	[118,119]
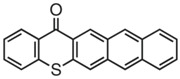 TX-A	λ_max_ ~ 368 ε_max_ ~ 14,000	[120,121]
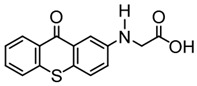 TX-NPG	λ_max_ ~ 392,583 ε_392nm_ ~ 1670 ε_583nm_ ~ 440	[124]
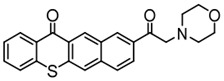 TX-MPM	λ_max_ ~ 410 ε_max_ ~ 4390	[126]
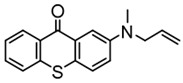 TX-1	λ_max_ ~ 438 ε_max_ ~ 4400	[127]
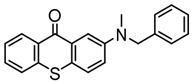 TX-2	λ_max_ ~ 438 ε_max_ ~ 4800	[127]
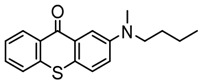 TX-3	λ_max_ ~ 444 ε_max_ ~ 4100	[127]
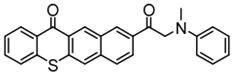 TX-MPA	λ_max_ ~ 407 ε_max_ ~ 3610	[128]
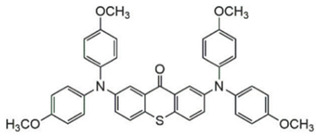 TX-2DPA	λ_max_ ~ 478 ε_max_ ~ 3400 ε_405nm_ ~ 2000	[129]
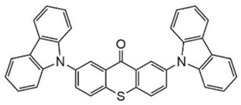 TX-2CBZ	λ_max_ ~ 396 ε_max_ ~ 7900 ε_405nm_ ~ 5900	[129]
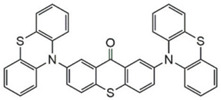 TX-2PTZ	λ_max_ ~ 305,415 ε_305nm_ ~ 2400 ε_405nm_ ~ 2600	[129]

## 4. Type Ⅰ Photoinitiators

Type Ⅰ PIs are used widely in industry and research laboratories. Oxime esters, acylphosphine oxides, and amino ketones are commonly used in photopolymerization. However, except for several commercial PIs, such as diphenyl(2,4,6-trimethylbenzoyl)phosphine oxide (TPO) and 1-benzyl-1-(dimethylamino)propyl 4-morpholinophenyl ketone (369), most of Type Ⅰ PIs do not absorb light above 400 nm. Therefore, it is important to improve the absorption ability of Type Ⅰ PIs to match the emission spectra of long-wavelength LEDs. Herein, oxime esters and acylphosphine oxides are mainly summarized in this review.

### 4.1. Oxime Ester Photoinitiators

Oxime esters have been investigated widely as Type Ⅰ PIs due to their high photoactivity [133,134]. Commercial oxime esters, OXE 01 and OXE 02, have been used to successfully produce thin films in color filter resists [135]. Under irradiation, the N−O bond in the oxime ester structure can break to produce iminyl and acyloxy radicals. The decarboxylation reaction occurs in the acyloxy radical to produce CO_2_ and in another active radical that can induce free radical polymerization. As the OXE 01 and OXE 02 can absorb UV light only, they perform poorly under visible light. Therefore, the design of novel oxime esters which can be used under visible LEDs is important. The chemical structures of oxime ester PIs mentioned in this review are shown in Table 8. Due to the different structures and substituents, the presented oxime esters exhibited very different light absorption properties, as shown in their maximal absorption wavelengths (λ_max_ in Table 8).

A series of oxime ester PIs based on the coumarin chromophore were synthesized by Li et al. [136]. They were designed to study the substituent and electronic effects. These PIs exhibited strong absorption, and the λ_max_ of O-3 and O-4 were 436 nm (ε_436nm_ = 41,690 M^−1^ cm^−1^) and 433 nm (ε_433nm_ = 7680 M^−1^ cm^−1^), respectively. Compared to O-4, O-3 demonstrated a dramatically increased molar extinction coefficient, which could be ascribed to the substitution position of the oxime-ester moiety. Stronger absorption of O-3 could be ascribed to the planar conformation and increased degree of conjugation [137]. The λ_max_ of O-3F and O-3O were all 436 nm. The photoinitiation ability of the PIs was investigated under a LED at 450 nm. O-3 demonstrated the best performance among them due to its strong absorption. Interestingly, both high-thiol and vinyl double-bond conversions were found using O-3 in thiol-based click polymerization. O-3 exhibit photobleaching property, and the curing depth was 2.6 mm within 10 min irradiation upon exposure to LED at 450 nm (200 mW/cm^2^). Indeed, the colorless photoreaction products allowed the light to penetrate deeper into the formulation. In addition, some oxime ester PIs based on coumarin chromophores were investigated and applied in 3D printing [138,139].

Hammoud et al. [140] designed some oxime esters based on the coumarin scaffold. All the chemical structures exhibited favorable molar extinction coefficients at 405 nm (i.e., OXE-D: ε_405nm_ = 22,500 M^−1^ cm^−1^; OXE-J: ε_405nm_ = 25,000 M^−1^ cm^−1^). The Type Ⅰ photoinitiation behaviors of the oxime esters were investigated upon exposure to LED at 405 nm in the FRP of TMPTA. OXE-J and OXE-D exhibited better initiation performances than the others, and the function conversions of TMPTA were 72% and 73%, respectively. According to computational calculations, the enthalpy value of the decarboxylation reaction and the cleavage process for the N–O bond was energetically favorable for OXE-D. In addition, the highest spin density of the methyl radical contributed to the good reactivity of OXE-D in polymerization experiments. Finally, a 3D-printed object was successfully obtained using an OXE-D/Iod system in acrylate monomers.

Two arylaminocarbazole oxime ester PIs (OXE1 and OXE2) were designed by Ma et al. [141]. In addition to Type Ⅰ PIs, OXE1 and OXE2 could also play the role of photosensitizer in multicomponent systems. OXE1 and OXE2 had wide absorption ranges at 200~450 nm. Under irradiation at 405 nm, the absorbance of OXE1 and OXE2 decreased to a certain extent. The results indicated the generation of stable photolysis products. When iodonium salt was added, the photolysis rate of the OXE1/iodonium salt system was faster than that of OXE1 alone. It suggested that the interaction of OXE1/iodonium salt was quick and efficient. The favorable values of free energy changes for OXE1/iodonium salt and OXE2/iodonium salt systems were obtained. In addition, the phenyl radical was detected in OXE1/iodonium salt system by spin-trapping ESR experiments. The FRP of TPGDA was studied upon exposure to the laser diode at 405 nm. TPGDA polymerized because of the oxime ester moiety in these structures. Higher function conversions were found using OXEs/iodonium salt (0.2%/1%, *w*/*w*). The photoinitiation performance was in line with the results of photolysis and the ESR experiments. The polymerization of epichlorohydrin was evaluated using OXEs/iodonium salt systems, and favorable function conversions were obtained. Indeed, the OXEs/iodonium salt systems could generate cations through electron transfer reactions to induce epoxide ring-opening polymerization. In conclusion, the arylaminocarbazole oxime esters broaden the application of oxime esters in visible light photopolymerization.

Ding et al. [142] designed two oxime esters named E-FBOXEs. The FRP of TPGDA was investigated upon exposure to LED at 395 nm. High function conversions of 81% and 80% were obtained using E-FBOXE-Me and E-FBOXE-ph, respectively. Interestingly, photochromism of the polymers prepared by E-FBOXEs was found under heating. After polymerization, a brown PTPGDA film was obtained using E-FBOXE-Me. The brown turned colorless when the film was heated at 50 °C. The photochromic mechanism was investigated by ESR and FTIR spectra. The results demonstrated that the N−O bond in E-FBOXE-Me molecules underwent cleavage under LED at 395 nm irradiation. Then, under heating, the generated acetoxy and colorful iminyl radicals could recombine to generate neutral E-FBOXE-Me structures. This special photochemical mechanism of E-FBOXEs makes it possible to design photochromic materials.

Four oxime esters based on carbazole-coumarin fused subunit were designed by Zhou et al. [143]. All the oxime esters exhibit similar absorption spectra with the maximum absorption wavelength λ_max_ = 374 nm. The absorbance of OXE-EM decreased gradually under LED at 365 nm irradiation. Meanwhile, this process was also monitored through ^1^H NMR. The results demonstrated the dissociation of the N-O bond. In addition, methyl radical was detected in spin-trapping ESR experiments. The FRP of TMPTA was evaluated by Photo-DSC. Under LED at 365, 385 and 405 nm irradiation, the photoinitiation ability of the oxime esters followed the order OXE-EM > OXE-IM > OXE-EP > OXE-IP. It was interesting that these PIs demonstrated better performance under LED at 405 nm than LED at 365 nm, although the molar extinction coefficients at 365 nm were higher. When irradiated at 365 or 405 nm, the photolysis behavior of OXE-EM was similar. A wavelength-dependent photopolymerization mechanism was proposed in this work. In addition, stilbene-based, phenyl thienyl thioether-based, and bicarbazole-based oxime ester PIs were also reported by Jin’s group [144,145,146].

Recently, three oxime esters based on the nitro-carbazole scaffold were designed (namely OXE-M, OXE-V, and OXE-P) [147]. Interestingly, OXE-M (methyl substituent) showed higher efficiency than OXE-P (phenyl substituent). It could be attributed to the decarboxylation reactions. Afterward, a series of oxime ester derivatives (i.e., D1, D2) with different substituents was designed to investigate the relationships between chemical structures and performances [148]. The light absorption properties (i.e., ε_405nm_ (**D1**) = 5200 M^−1^ cm^−1^, ε_405nm_ (**D2**) = 5400 M^−1^ cm^−1^) are good to use a LED at 405 nm. Interestingly, some PIs had better performance than TPO under LED at 405 nm irradiation. The effect of the substituents was studied by theoretical calculations, monitoring of CO_2_, and the study of free radicals. All results show that substituents have an effect on the performance of PIs in polymerization experiments via a decarboxylation reaction.

**Table 8:** The chemical structures and light absorption properties of oxime ester photoinitiators.

Structure	Absorption Properties (λ/nm, ε_max_/M^−1^ cm^−1^)	Refs.
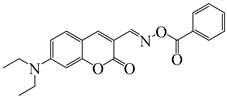 O-3	λ_max_ ~ 436 ε_max_ ~ 41,690 ε_450nm_ ~ 37,450	[136]
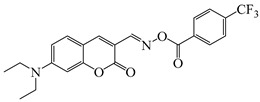 O-3F	λ_max_ ~ 436 ε_max_ ~ 29,930 ε_450nm_ ~ 26,630	[136]
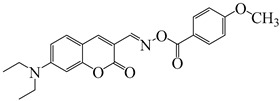 O-3O	λ_max_ ~ 436 ε_max_ ~ 29,950 ε_450nm_ ~ 26,620	[136]
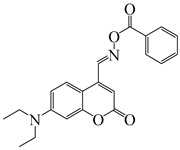 O-4	λ_max_ ~ 433 ε_max_ ~ 7680 ε_450nm_ ~ 6790	[136]
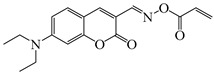 DCCA	λ_max_ ~ 436 ε_max_ ~ 51,000 ε_450nm_ ~ 45,000	[138]
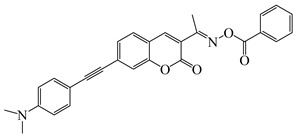 OEC3–1	λ_max_ ~ 406 ε_max_ ~ 51,000 ε_450nm_ ~ 21,000	[139]
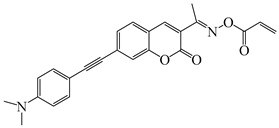 OEC3–2	λ_max_ ~ 405 ε_max_ ~ 42,000 ε_450nm_ ~ 17,000	[139]
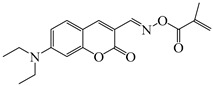 OXE-B	λ_max_ ~ 431 ε_max_ ~ 33,000 ε_405nm_ ~ 22,000	[140]
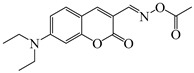 OXE-D	λ_max_ ~ 431 ε_max_ ~ 34,000 ε_405nm_ ~ 22,500	[140]
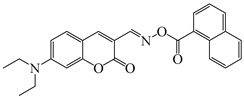 OXE-E	λ_max_ ~ 437 ε_max_ ~ 28,500 ε_405nm_ ~ 16,500	[140]
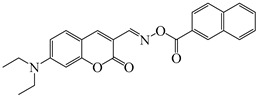 OXE-F	λ_max_ ~ 437 ε_max_ ~ 36,000 ε_405nm_ ~ 21,000	[140]
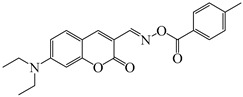 OXE-G	λ_max_ ~ 436 ε_max_ ~ 31,500 ε_405nm_ ~ 18,000	[140]
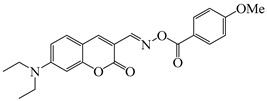 OXE-H	λ_max_ ~ 435 ε_max_ ~ 26,500 ε_405nm_ ~ 17,000	[140]
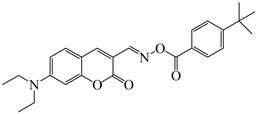 OXE-I	λ_max_ ~ 437	[140]
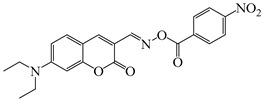 OXE-J	λ_max_ ~ 441 ε_max_ ~ 50,000 ε_405nm_ ~ 25,000	[140]
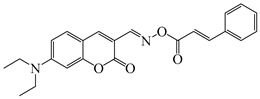 OXE-K	λ_max_ ~ 435 ε_max_ ~ 31,000 ε_405nm_ ~ 18,000	[140]
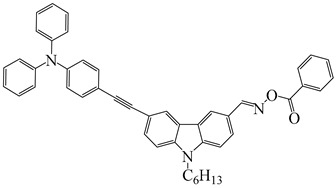 OXE1	λ_max_ ~ 350 ε_max_ ~ 46,900 ε_405nm_ ~ 1100	[141]
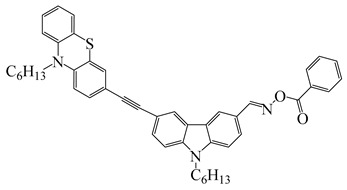 OXE2	λ_max_ ~ 357 ε_max_ ~ 29,300 ε_405nm_ ~ 4410	[141]
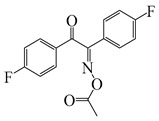 E-FBOXE-Me	λ_max_ ~ 260 ε_260nm_ ~ 17,980 ε_395nm_ ~ 20	[142]
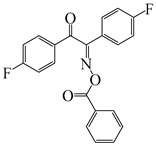 E-FBOXE-Ph	λ_max_ ~ 262 ε_260nm_ ~ 51,410 ε_395nm_ ~ 50	[142]
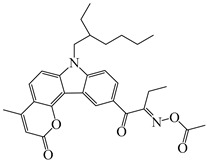 OXE-EM	λ_max_ ~ 374 ε_max_ ~ 16,400 ε_355nm_ ~ 12,500	[143]
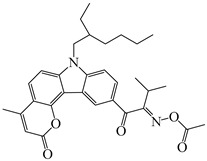 OXE-IM	λ_max_ ~ 374 ε_max_ ~ 16,000 ε_355nm_ ~ 12,800	[143]
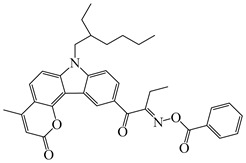 OXE-EP	λ_max_ ~ 374 ε_max_ ~ 17,000 ε_355nm_ ~ 13,200	[143]
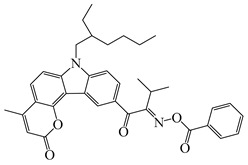 OXE-IP	λ_max_ ~ 374 ε_max_ ~ 16,600 ε_355nm_ ~ 13,000	[143]
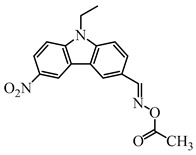 OXE-M	λ_max_ ~ 369 ε_max_ ~ 13,000 ε_405nm_ ~ 4100	[147]
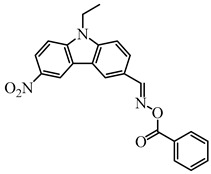 OXE-P	λ_max_ ~ 368 ε_max_ ~ 13,800 ε_405nm_ ~ 4100	[147]
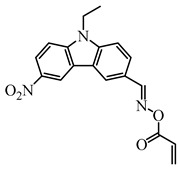 OXE-V	λ_max_ ~ 369 ε_max_ ~ 12,400 ε_405nm_ ~ 3900	[147]
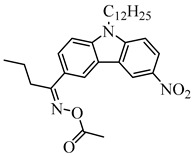 D1	λ_max_ ~ 372 ε_max_ ~ 13,000 ε_405nm_ ~ 5200	[148]
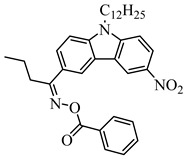 D2	λ_max_ ~ 372 ε_max_ ~ 14,000 ε_405nm_ ~ 5400	[148]

### 4.2. Acylphosphine Oxide Photoinitiators

Acylphosphine oxides are another class of Type Ⅰ PI. They are widely used in many fields due to their high efficiency and good photobleaching. As the absorption of most acylphosphine oxide PIs is located in the UV range, it is important to broaden their absorption of them in the visible range to match the emission spectra of LEDs. The chemical structures of acylphosphine oxide PIs mentioned in this review are shown in Table 9.

A series of acylphosphine oxide compounds were proposed by Dietlin et al. [149]. Before synthesis, molecular modeling was used to select the potentially reactive compounds. The maximum absorption wavelength was computed to match the selected LEDs. The energy of the triplet state (E_T_), as well as the bond dissociation energy (BDE), were computed to explore whether the cleavage of the bond was energetically favorable (⊗H < 0). The spin density on the radical center was computed to ensure the efficient addition of the double bond; 7 molecules were selected from 86 molecules based on favorable calculated parameters. The molar extinction coefficients at 395 nm of acylphosphine oxide compounds (except ADPO-6 and ADPO-7) were higher than that of TPO-L. The FRP of TMPTA was performed under LED at 395 nm irradiation using (1 wt%). All the photoinitiators (except ADPO-5) demonstrated higher final function conversions than commercial TPO-L and BAPO. Although ADPO-5 had good absorption properties, the cleavage of the bond took place with difficulty. The rational design of acylphosphine oxide compounds is helpful in selecting an efficient PI to use in LED photopolymerization.

An acylphosphine oxide PI 4-(diethylamino)benzoyldiphenylphosphine oxide (DEAPO) was designed and synthesized by Xie et al. [150]. Compared to TPO (λ_max_ = 380 nm), a red-shifted maximum absorption wavelength was obtained for DEAPO (λ_max_ = 386 nm). Interestingly, the absorption band of DEAPO extended to 440 nm and high molar extinction coefficients of DEAPO was obtained (ε_385nm_ = 43,800 M^−1^ cm^−1^; ε_420nm_ = 5950 M^−1^ cm^−1^). The good absorption property was ascribed to the diethyl amino moiety in the DEAPO structure (prolonging the conjugation system). DEAPO was evaluated as a Type Ⅰ PI with TMPTA. Upon exposure to LED at 385 nm, the function conversion of the DEAPO system (FC = 68.7%) was higher than the TPO system (FC = 58.2%). In addition, the DEAPO system (FC = 64.3%) demonstrated better performance than the TPO system (FC = 47.1%) under LED at 420 nm irradiation. Indeed, good absorption properties for DEAPO allowed the efficient initiation ability under LEDs. The results demonstrated that DEAPO has the potential to be applied in the field of dental materials or food packaging.

Two carbazolyl-based acylphosphine oxide compounds (ETPO and ALPO) were synthesized by Wu et al. [151]. Favorable molar extinction coefficients were observed for ETPO (ε_405nm_ = 2270 M^−1^ cm^−1^) and ALPO (ε_405nm_ = 1300 M^−1^ cm^−1^), which could be attributed to the large rigid plane and the strong conjugate system in carbazole moiety. In steady-state photolysis experiments, the absorbance peak of ETPO and ALPO decreased dramatically under LED at 395 nm irradiation, which exhibit high photoactivity for the two compounds. The FRP of TMPTA (~40 μm) was studied upon exposure to LED at 405 nm. Acrylate function conversions for monomers were 44.7%, 75.7%, and 56.7% using ETPO, ALPO and TPO (1 wt%), respectively. Due to the bad solubility in monomers, the efficiency of ETPO in TMPTA was low. ALPO demonstrated better performance than TPO due to its high photoactivity and good absorption properties. ALPO can be used as a visible PI in many fields.

**Table 9:** The chemical structures and light absorption properties of acylphosphine oxide photoinitiators.

Structure	Absorption Properties (λ/nm, ε_max_/M^−1^ cm^−1^)	Refs.
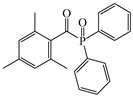 TPO	λ_max_ ~ 380 ε_max_ ~ 570 ε_385nm_ ~ 510 ε_420nm_ ~ 20	[150]
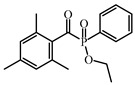 TPO-L	λ_max_ ~ 383 ε_395nm_ ~ 130	[149]
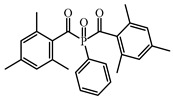 BAPO	λ_max_ ~ 384 ε_395nm_ ~ 660	[149]
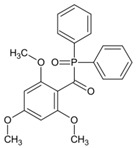 ADPO-1	λ_max_ ~ 373 ε_395nm_ ~ 300	[149]
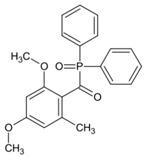 ADPO-2	λ_max_ ~ 379 ε_395nm_ ~ 390	[149]
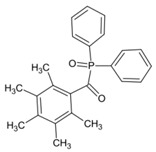 ADPO-3	λ_max_ ~ 382 ε_395nm_ ~ 1060	[149]
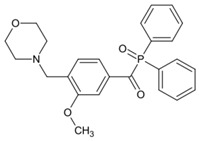 ADPO-4	λ_max_ ~ 386 ε_395nm_ ~ 160	[149]
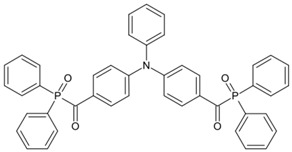 ADPO-5	λ_max_ ~ 405 ε_395nm_ ~ 26,600	[149]
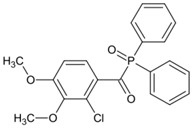 ADPO-6	λ_max_ ~ 405	[149]
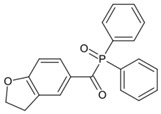 ADPO-7	λ_max_ ~ 384	[149]
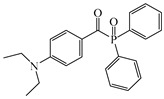 DEAPO	λ_max_ ~ 386 ε_max_ ~ 43,810 ε_385nm_ ~ 43,800 ε_420nm_ ~ 5950	[150]
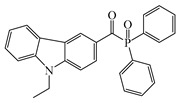 ETPO	λ_max_ ~ 366 ε_max_ ~ 13,830 ε_395nm_ ~ 4530 ε_405nm_ ~ 2270	[151]
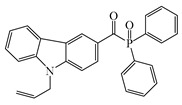 ALPO	λ_max_ ~ 362 ε_max_ ~ 12,200 ε_395nm_ ~ 2890 ε_405nm_ ~ 1300	[151]

### 4.3. Other Type I photoinitiators

An acylstannane-based PI tetrakis(2,4,6-trimethylbenzoyl)stannane (see Table 10) was designed for visible light photopolymerization [152]. Acylstannanes could show Type I PIs features like those in acylgermanes [153,154,155]. The absorption band of this acylstannane can extend to 550 nm. In steady-state photolysis, fast photobleaching was observed for the acylstannane (1 × 10^−3^ mol/L in acetonitrile) with a LED at 460 nm (1 W/cm^2^) after 3 min. It was attributed to the cleavage of the Sn-CO bond. The photoinitiation ability was studied under LED at 460 and LED at 522 nm. Upon exposure to a LED at 460 nm, tetrakis(2,4,6-trimethylbenzoyl)stannane demonstrated a similar performance with Ivocerin^®^, and high acrylate conversion was found. In addition, the acylstannane still showed high reactivity under LED at 522 nm. Interesting, favorable curing depths were obtained in the presence of this acylstannane-based PI. In prospect, it can be applied in many industrial fields.

Some N-hydroxynaphthalimide ester derivatives (namely NPIE1–NPIE9, see Table 10) are proposed as Type I PIs [156]. They demonstrated good light absorption properties, such as ε_405nm_ (NPIE1) = 15,000 M^−1^ cm^−1^ and ε_405nm_ (NPIE2) = 14,400 M^−1^ cm^−1^. Interestingly, these PIs demonstrated a favorable performance for the FRP of the monomers. NPIE1 (FC = 68%) demonstrated better performance than benchmark structure TPO (FC = 66%) in polymerization experiments of TMPTA. A decarboxylation process was discovered for these structures. The mechanisms N-hydroxynaphthalimide ester derivatives were investigated using a computational procedure, steady-state photolysis, and fluorescence approaches. Finally, the cleavage mechanism (N−O bond) of the Type Ⅰ PI was proposed.

**Table 10:** The chemical structures and light absorption properties of other Type I photoinitiators.

Structure	Absorption Properties (λ/nm, ε_max_/M^−1^ cm^−1^)	Refs.
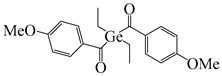 Ivocerin^®^	λ_max_ ~ 408 ε_max_ ~ 711 ε_385nm_ ~ 505	[157]
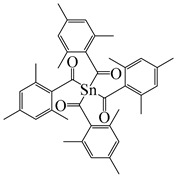 tetrakis(2,4,6-trimethylbenzoyl)stannane		[152]
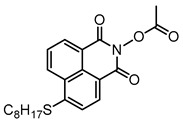 NPIE1	λ_max_ ~ 397 ε_max_ ~ 16,000 ε_405nm_ ~ 15,000	[156]
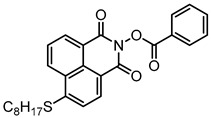 NPIE2	λ_max_ ~ 398 ε_max_ ~ 15,200 ε_405nm_ ~ 14,400	[156]
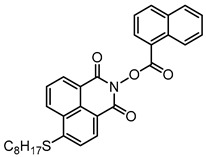 NPIE3	λ_max_ ~ 398 ε_max_ ~ 15,600 ε_405nm_ ~ 14,900	[156]
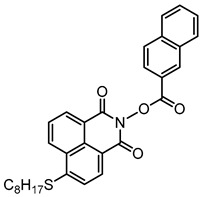 NPIE4	λ_max_ ~ 397 ε_max_ ~ 14,100 ε_405nm_ ~ 13,100	[156]
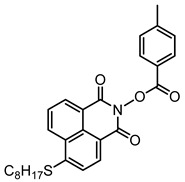 NPIE5	λ_max_ ~ 397 ε_max_ ~ 15,300 ε_405nm_ ~ 14,500	[156]
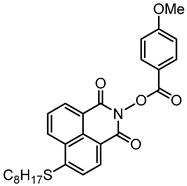 NPIE6	λ_max_ ~ 397 ε_max_ ~ 14,700 ε_405nm_ ~ 13,800	[156]
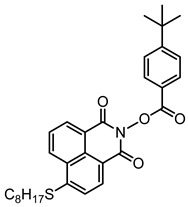 NPIE7	λ_max_ ~ 397 ε_max_ ~ 13,500 ε_405nm_ ~ 12,800	[156]
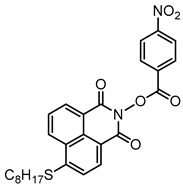 NPIE8	λ_max_ ~ 397 ε_max_ ~ 12,900 ε_405nm_ ~ 12,000	[156]
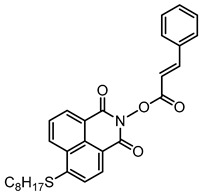 NPIE9	λ_max_ ~ 397 ε_max_ ~ 14,900 ε_405nm_ ~ 14,000	[156]

## 5. Conclusions and Perspectives

In the past few years, photoinitiators for LED photopolymerization have received increasing attention. An increasing number of organic dyes have been used in photoinitiating systems due to their good light absorption properties. Many chemical structures demonstrate good performance in FRP and CP under LED irradiation. The modification for traditional Type I and Type II photoinitiators have also been carried out frequently to expand the range of their applications. For the development of photoinitiators, besides light absorption properties, synthesis procedures, toxicity, and cost should also be taken into account. All the presented PIs remain on the lab scale even if industrial development can be considered. In the future, more efforts should be devoted to addressing biocompatibility, which can provide significant opportunities for photoinitiators in the field of biomaterials and biomedicine. In addition, it is also desirable and crucial to resolving the yellow coloration induced by some photoinitiators during the photopolymerization. Some scaffolds with photo-bleaching characteristics are a potential option for the design of photoinitiators with a favorable curing depth.

## Data Availability

The data presented in this study are available on request from the corresponding author.
